# Conservation recommendations for *Oryza rufipogon* Griff. in China based on genetic diversity analysis

**DOI:** 10.1038/s41598-020-70989-w

**Published:** 2020-09-01

**Authors:** Junrui Wang, Jinxia Shi, Sha Liu, Xiping Sun, Juan Huang, Weihua Qiao, Yunlian Cheng, Lifang Zhang, Xiaoming Zheng, Qingwen Yang

**Affiliations:** 1 Institute of Crop Sciences, Chinese Academy of Agricultural Sciences, Beijing, China; 2 Shanghai Normal University, Shanghai, China; 3 Shanxi Agricultural University, Jinzhong, China; 4 Institute of Rice Research, Guangxi Academy of Agricultural Sciences, Nanning, China; 5 Agricultural Science and Technology Innovation Program/Crop Germplasm Resources Preservation and Sharing Innovation Team, Beijing, China

**Keywords:** Agricultural genetics, Evolutionary biology

## Abstract

Over the past 30 years, human disturbance and habitat fragmentation have severely endangered the survival of common wild rice (*Oryza rufipogon* Griff.) in China. A better understanding of the genetic structure of *O. rufipogon* populations will therefore be useful for the development of conservation strategies. We examined the diversity and genetic structure of natural *O. rufipogon* populations at the national, provincial, and local levels using simple sequence repeat (SSR) markers. Twenty representative populations from sites across China showed high levels of genetic variability, and approximately 44% of the total genetic variation was among populations. At the local level, we studied fourteen populations in Guangxi Province and four populations in Jiangxi Province. Populations from similar ecosystems showed less genetic differentiation, and local environmental conditions rather than geographic distance appeared to have influenced gene flow during population genetic evolution. We identified a triangular area, including northern Hainan, southern Guangdong, and southwestern Guangxi, as the genetic diversity center of *O. rufipogon* in China, and we proposed that this area should be given priority during the development of ex situ and in situ conservation strategies. Populations from less common ecosystem types should also be given priority for in situ conservation.

## Introduction

Common wild rice (*Oryza rufipogon* Griff.) is the putative progenitor of Asian cultivated rice, one of the most important food crops in the world. It is also an important source of germplasm for rice improvement^1–3^. Ding Ying found wild rice (*O. rufipogon*) in Guangzhou in 1926, and the wild × cultivated cross Zhong Shan No. 1 was widely planted in South China for more than 50 years. In 1970, Yuan Longping and his assistant discovered wild rice with male sterility in Hainan and used it to breed high yielding “three-line” hybrid rice varieties^4,5^. The increased yield of this hybrid rice saved thousands of lives in China and around the world. In recent years, wild rice has been used to introduce genes that confer agriculturally beneficial traits into cultivated species, and it holds great potential for future rice breeding efforts.

Before the 1970s, *O. rufipogon* was found in 113 counties of eight provinces in southern China, including Guangdong, Guangxi, Hainan, Yunnan, Hunan, Jiangxi, Fujian, and Taiwan, although the populations in Taiwan disappeared in 1978^6,7^. Since the 1980s, many wild rice habitats have been converted to agricultural or industrial use because of the rapid development of the rural economy and the population expansion in rural China. Consequently, areas of wild rice cultivation have dramatically decreased. Our recent work indicates that approximately 70% of the *O. rufipogon* populations have disappeared, and all large populations (growth area > 33 hm^2^) have either disappeared or decreased dramatically (unpublished data).

The threatened status of wild rice has attracted increasing attention in China, and there is a desire to collect samples for ex situ conservation and to develop in situ conservation programs. The Chinese government began to invest in the construction of in situ conservation sites in 2001, and 15 such sites were established by the end of 2014. The government also established eight other in situ conservation sites using a mainstreaming approach, a new conservation strategy in which farmers are encouraged to participate in conservation activities that span physical boundaries such as hills and rivers. Most in situ conservation sites were selected based on scientific expertise or local government recommendations, although it was still necessary to justify their value and rationale. Detailed information on the population genetic structure of *O. rufipogon* is therefore useful to guide the selection of future sites.

Information generated using molecular methods has direct and indirect consequences for the practical management and conservation of germplasm. Genetic diversity data can be useful for understanding the taxonomy and evolution of crop species, and this basic knowledge supports their conservation^8^. More directly, genetic diversity studies can help us to adjust our strategies for collection, evaluation, and breeding. Although some studies have recently documented the population genetic structure of *O. rufipogon* in China^9–13^, few have focused on the development of conservation strategies based on this population genetic structure. Our study used SSR markers to examine the genetic diversity and population genetic structure of natural populations at three different levels: national (China), provincial (Guangxi Province), and local (Dongxiang population in Jiangxi Province).

## Results

### Genetic diversity of *O. rufipogon* populations

**Figure 1:**
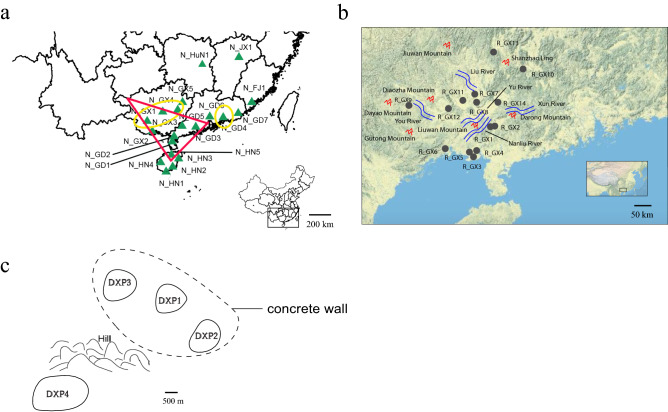
Locations of natural *O. rufipogon* populations used in this study **(a)** Map of 20 *O. rufipogon* populations in China generated by R version 3.5.2. The red triangular area represents the genetic diversity center identified in the present study, and the yellow ovals indicate the diversity center reported by Wang^58^. **(b)** The locations of 14 populations from Guangxi (URL: https://commons.wikimedia.org/wiki/File: China_topography_full_res.jpg). **(c)** The locations of populations from Dongxiang.

Twenty-four SSR primer pairs from previous studies^14^ with polymorphisms and a uniform distribution among chromosomes were selected for use in the analysis of population genetic diversity and genetic structure (Supplementary Table S1). All loci were found to be in Hardy–Weinberg equilibrium. High levels of genetic variability at 24 loci were detected in 628 individuals from 20 populations sampled across China (Table 1; Fig. 1a). A total of 340 alleles were detected across the loci, ranging from 23 alleles at RM253 to seven alleles at RM244 and RM345 (Supplementary Table S2). The average number of alleles was 14.17. The overall means of *A*_E_ (the effective number of alleles), *H*_O_ (the observed heterozygosity), *H*_E_ (the expected heterozygosity) and *I* (the Shannon–Weaver information index) across all loci were 6.97, 0.58, 0.83, and 2.08, respectively. The values varied widely among loci: *A*_E_ ranged from 2.80 (RM244) to 15.41 (RM336); *H*_O_ ranged from 0.15 (RM244) to 0.88 (RM336); *H*_E_ ranged from 0.64 (RM244) to 0.93 (RM253); and *I* ranged from 1.13 (RM244) to 2.85 (RM336).

**Table 1:** Population codes, geographical localities, sample sizes and genetic diversity parameters of all *O. rufipogon* populations.

Population code	Population locality	Sample size	*A*	*Ae*	*He*	*Ho*	*I*	Private alleles
N_HN1	Sanya County, Hainan	7	1.57	1.54	0.27	0.53	0.38	0
N_HN2	Qionghai County, Hainan	38	2.29	1.8	0.39	0.7	0.57	1
N_HN3	Wenchang County, Hainan	43	1.89	1.67	0.34	0.66	0.48	1
N_HN4	Chengmai County, Hainan	24	2.54	1.91	0.44	0.81	0.66	0
N_HN5	Qiongshan County, Hainan	52	5.71	2.84	0.58	0.75	1.14	11
N_GD1	Leizhou County,Guangdong	6	2.57	1.8	0.38	0.42	0.64	1
N_GD2	Suixi County, Guangdong	31	4.61	2.75	0.59	0.65	1.1	4
N_GD3	Enping County, Guangdong	21	4.82	2.91	0.6	0.6	1.15	2
N_GD4	Huiyang County, Guangdong	19	3.39	1.83	0.4	0.42	0.72	2
N_GD5	Gaozhou County, Guangdong	30	2.5	1.78	0.39	0.69	0.59	1
N_GD6	Zengcheng County, Guangdong	11	1.82	1.59	0.3	0.53	0.44	1
N_GD7	Huilai County, Guangdong	32	4.86	2.31	0.5	0.53	0.97	1
N_GX1	Fusui County, Guangxi	33	4.04	2.48	0.56	0.67	1.01	0
N_GX2	Fangchenggang County, Guangxi	46	4.96	3.01	0.63	0.72	1.19	6
N_GX3	Wuxuan County, Guangxi	63	6.4	2.66	0.54	0.51	1.12	12
N_GX4	Hezhou County, Guangxi	45	4.46	2.58	0.5	0.56	0.95	1
N_GX5	Zhongshan County, Guangxi	25	3.61	2.18	0.45	0.33	0.83	5
N_FJ1	Zhangpu County, Fujian (conservation site)	11	3.04	1.89	0.4	0.43	0.7	1
N_HuN1	Chaling County, Hunan (conservation site)	35	4.93	2.58	0.53	0.36	1.04	0
N_JX1	Dongxiang County, Jiangxi (conservation site)	56	4	2.25	0.49	0.44	0.88	1
Means	Populations from the diversity center without N_GD1		4.72**	2.63**	0.56**	0.66**	1.04**	
	Populations from the diversity center		4.45*	2.53**	0.53**	0.63**	0.99*	
	Populations from the whole country		3.7	2.22	0.46	0.57	0.83	

*A* mean number of alleles per locus, *Ae* effective number of alleles, *He* expected heterozygosity, *Ho* observed heterozygosity, *I* Shannon–Weaver information index.

*P < 0.05; **P < 0.01.

The genetic structure of populations across China was analyzed. The mean value of *F*_ST_ was 0.44, and it varied from 0.25 to 0.59, indicating that there was substantial genetic variation among populations (Supplementary Table S2). Genetic population differentiation was also measured by AMOVA analysis (Table 2) and pairwise population differentiation (Supplementary Table S3). AMOVA analysis showed that 41.2% of the variation occurred among populations. The significant differentiation (the *P* value of *F*_ST_ < 0.001) among populations also reflected larger differences between all populations from the whole country (Table 2). *F*_IS_ ranged from − 0.49 to 0.44 with an average of − 0.20, consistent with the complex mating system of this species. Many studies have suggested that the mating system of *O. rufipogon* is a mix of outcrossing and selfing^15^. This result indicated that most populations deviated from the Hardy–Weinberg expectation within populations and had an excess of heterozygotes. The genetic diversity parameters of natural *O. rufipogon* populations in China were also summarized by province (Table 3). Among the provinces, Guangxi showed the highest levels of genetic diversity with *A*_E_ = 5.14, *H*_O_ = 0.77 and *I* = 1.80. The next highest levels of genetic diversity were found in Guangdong, Hainan, Hunan, Jiangxi, and Fujian. The correlation between *F*_*ST*_ and geographic distance in Chinese *O. rufipogon* populations was nearly significant (*p* = 0.07874).

**Table 2:** AMOVA results for twenty populations in China.

Source of variation	df	Sum of squares	Variance components	Percentage of variation	*P* value
Among populations	19	5,883.6	4.9	41.2	< 0.001
Within populations	1,236	8,632.0	7.0	58.8	< 0.001
Total	1,255	14,515.6	11.9

**Table 3:** Genetic diversity parameters of natural *O. rufipogon* populations in China by province.

Population	*A*	*Ae*	*He*	*Ho*	*I*
Hainan	8.18	4.44	0.71	0.75	1.61
Guangxi	10.75	5.14	0.57	0.77	1.80
Guangdong	10.29	4.99	0.58	0.76	1.76
Fujian	3.04	1.89	0.43	0.40	0.70
Hunan	4.93	2.58	0.36	0.53	1.04
Jiangxi	4.00	2.25	0.44	0.49	0.88

*A* mean number of alleles per locus, *Ae* effective number of alleles, *He* expected heterozygosity, *Ho* observed heterozygosity, *I* Shannon–Weaver information index.

### Genetic structure of *O. rufipogon* populations in China

**Figure 2:**
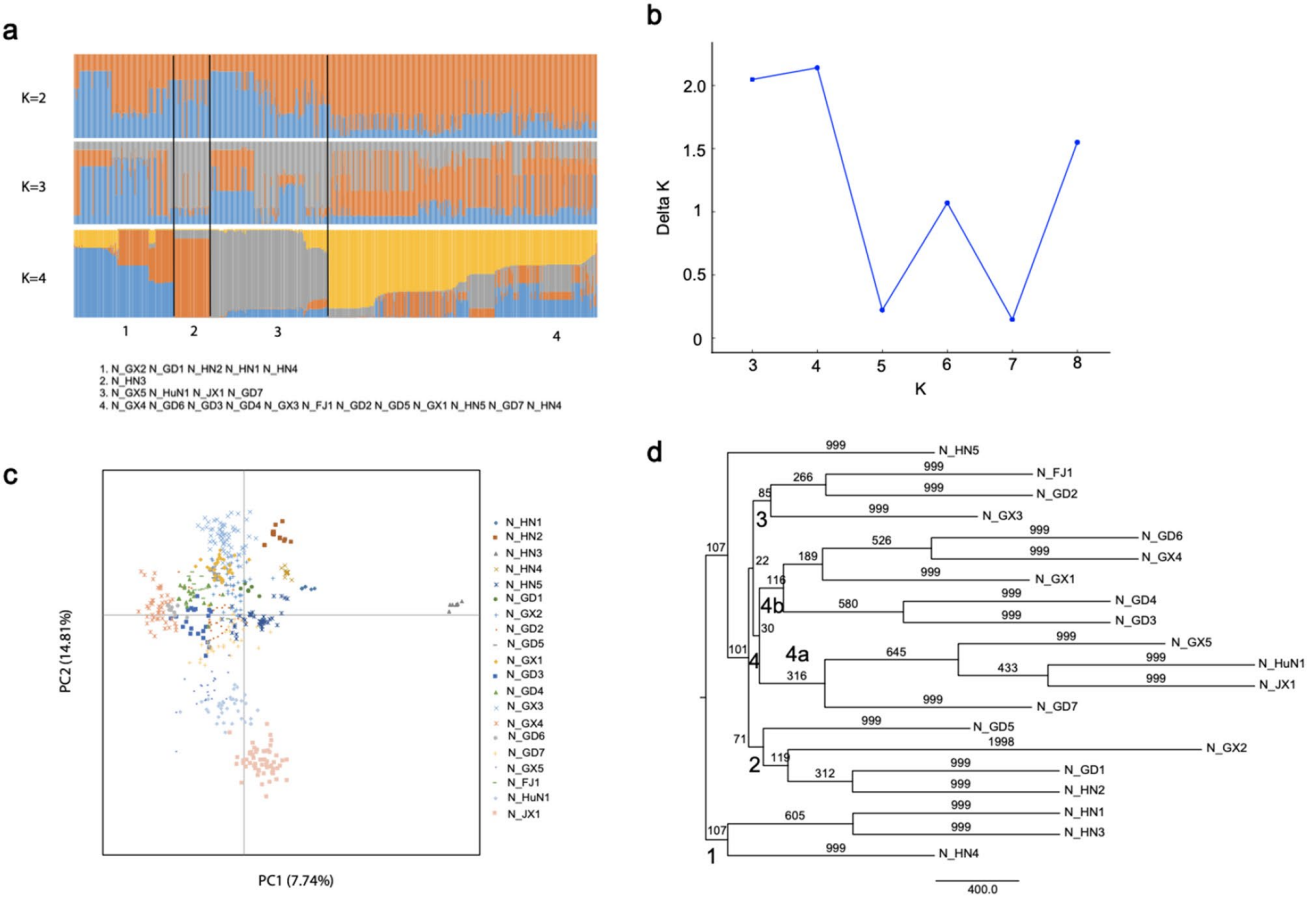
Population structure analysis of 20 natural *O. rufipogon* populations in China. **(a)** Clustering of 20 populations inferred with STRUCTURE (*K* = 2–4). **(b)** Delta *K* values for STRUCTURE analysis. **(c)** Results of principal component analysis. **(d)** UPGMA dendrograms based on Nei’s distance.

The genetic relationships among populations in China were analyzed with a population structure analysis in STRUCTURE, principal component analysis (PCA) and construction of a UPGMA tree (Fig. 2). In the structure analysis, the log-likelihood ln(*P*(*D*)) was largest when the number of populations, *K*, was equal to four (Fig. 2b), and four groups were therefore identified. Most populations from Hainan were placed in Group 1, together with two southern boundary populations N_GX2 and N_GD1. The N_HN3 population was placed by itself in Group 2. Two northern boundary populations from Guangdong and Guangxi were placed in Group 3 with N_HuN1 and N_JX1. Most populations from Guangdong and Guangxi were placed in Group 4, together with the northernmost population, N_HN5. Results of the PCA were consistent with those of STRUCTURE (Fig. 2c). The UPGMA dendrogram showed that the 20 populations formed three main clusters with a genetic similarity of approximately 0.27 (Fig. 2d). Three populations (N_HN1, N_HN3, and N_HN4) from Hainan were grouped into Cluster 1. N_HN2 from Hainan and N_GD1 and N_GD5 from Guangdong and N_GX2 from Guangxi were grouped into Cluster 2. Group 3 from the structure analysis corresponded to Cluster 4a, Group 4 from the structure analysis corresponded to Cluster 3 and Cluster 4b. Surprisingly, the two Hainan populations (N_HN2 and N_HN5) were placed in different clusters rather than forming a separate cluster. N_HN2 was placed in Cluster 2 with populations from Guangdong and Guangxi, and N_HN5 was separated away from the others.

**Figure 3:**
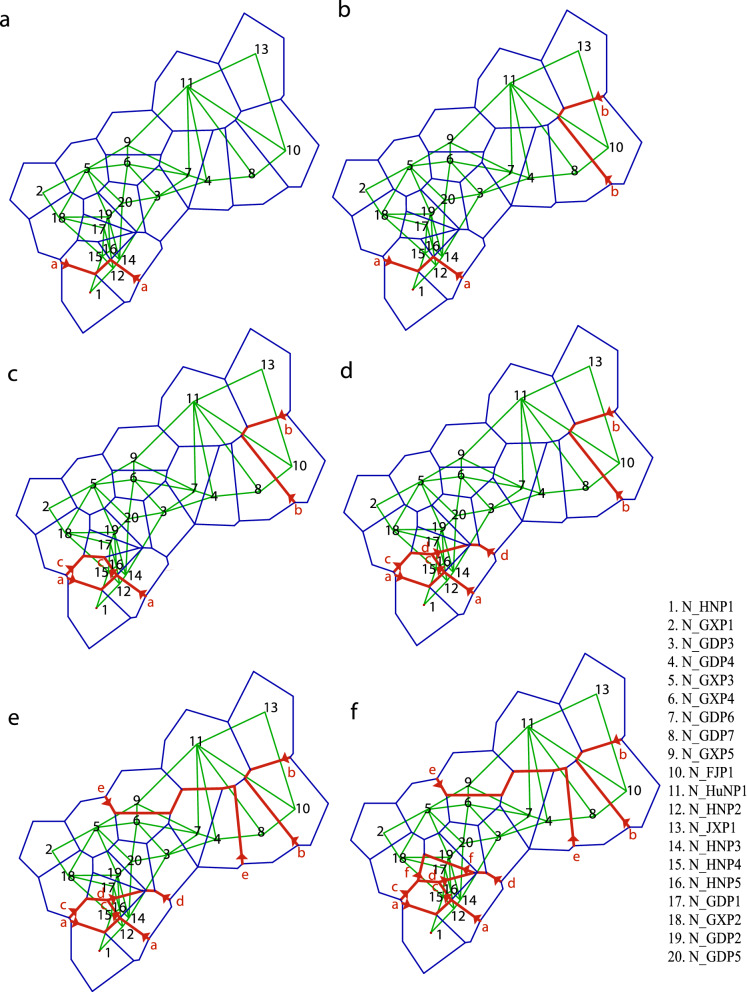
Successive genetic barriers predicted with BARRIER software. The genetic barriers are shown as bold red lines with arrows. Lines a–f indicate genetic barriers, and alphabetical order indicates the sequence of boundary formation.

Monmonier’s maximum difference algorithm was used to perform a genetic barrier prediction analysis with all populations included (Fig. 3). The first predicted barrier separated N_HN1 and N_HN2 from all other populations. The second barrier separated population N_FJ1, which was located in the easternmost sampled area. The third predicted barrier separated N_HN4, and the fourth barrier separated N_HN3 and N_HN5. Cluster 3b (Group 3) was separated by the fifth barrier, and N_GD1 by the sixth barrier.

**Figure 4:**
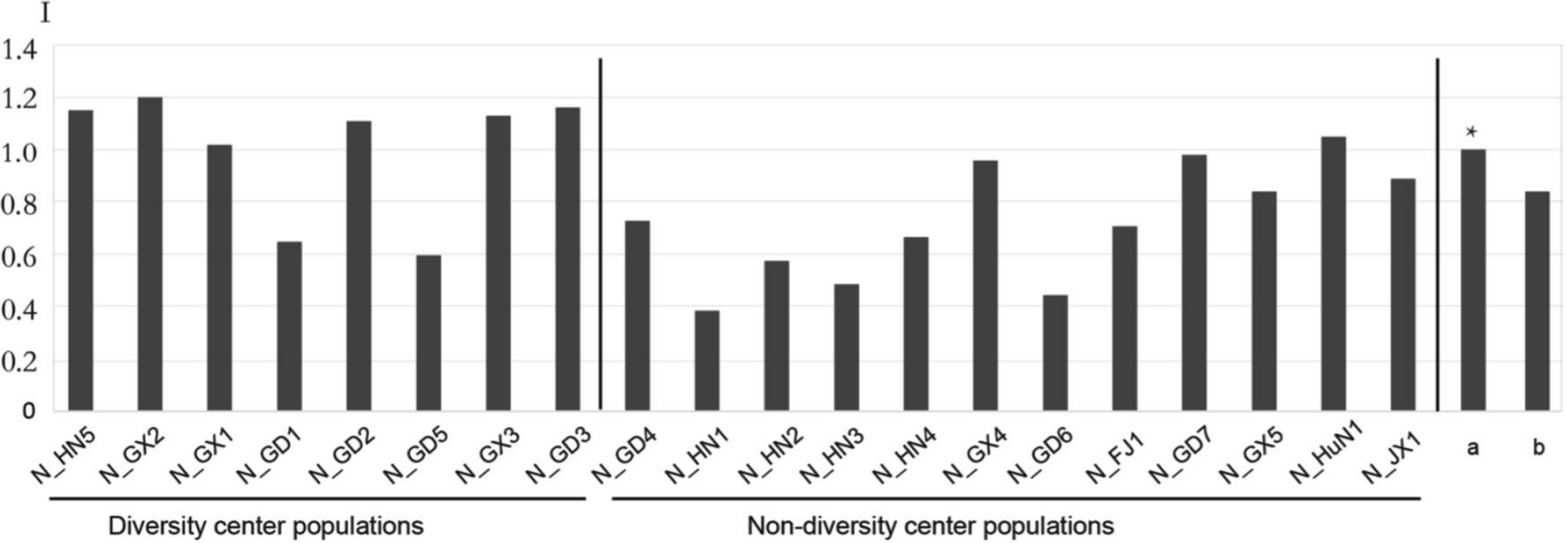
Comparison of genetic diversity parameters among 20 natural populations and between groups within and outside the genetic diversity center. **(a)** Mean for populations in the diversity center. **(b)** Mean for populations from across the whole country.

The populations with higher genetic diversity formed a triangular area (Fig. 1a) that reached from 19° N to 23° N and included northern Hainan, southern Guangdong, and southwestern Guangxi. It included the N_GX1, N_GX3, and N_GX2 populations from Guangxi, the N_GD1, N_GD2, N_GD5, and N_GD3 populations from Guangdong, and the N_HN5 population from Hainan. As shown in Table 1, the averages of the diversity parameters in this triangular area were significantly higher than those of other populations and higher than the overall population averages (Fig. 4). The higher diversity and greater number of private alleles indicate that this triangular area may be the genetic diversity center of *O. rufipogon* in China.

### Population structure and differentiation of *O. rufipogon* in local regions

To understand the history of wild rice populations, we conducted an in-depth study of the largest (Guangxi) (Table 4) and smallest (Jiangxi) populations of wild rice in China. The values of the within-population genetic diversity (*H*_S_), the among-population genetic diversity (*D*_ST_), and the coefficient of genetic differentiation (*G*_ST_) were 0.38, 0.36 and 0.49, respectively, suggesting that most genetic variability occurred among populations, although *G*_ST_ showed somewhat higher differentiation than *D*_ST_. These results agreed with the tests of pairwise genetic identity among populations (Supplementary Table S4).

**Table 4:** Population codes, geographical localities, sample sizes and genetic diversity parameters of all *O. rufipogon* populations in Guangxi province.

Population code	Population locality	Sample size		*A*	*Ae*	*He*	*Ho*	*I*
R_GX1	Bobai County, Yulin	30		4.97	3.4	0.62	0.66	1.27
R_GX2	Fumian Ditrict, Yulin	29		4.31	2.74	0.63	0.59	1.09
R_GX3	Yinhai District, Beihai	26		3.33	2.05	0.62	0.44	0.74
R_GX4	Tieshangang District, Beihai	25		4.25	2.91	0.57	0.6	1.11
R_GX5	Hepu County, Beihai	30		5.19	2.77	0.55	0.58	1.09
R_GX6	Fangcheng District, Fangchenggang	29		4	2.46	0.55	0.51	0.94
R_GX9	Tiandong County, Baise	27		3.33	2.07	0.44	0.4	0.77
R_GX10	Zhongshan County, Hezhou	28		2	1.49	0.42	0.25	0.38
R_GX8	Wuxuan County, Laibin	30		4.33	2.34	0.4	0.49	0.91
R_GX11	Xingbin District, Laibin	30		4.61	2.31	0.31	0.49	0.93
R_GX7	Xiangzhou County, Laibin	28		4.56	2.89	0.46	0.56	1.06
R_GX14	Pingnan County, Guigang	25		3.03	2.12	0.38	0.48	0.8
R_GX12	Shanglin County, Nanning	23		2.83	1.99	0.37	0.38	0.64
R_GX13	Yanshan District, Guilin	20		3.58	2.21	0.36	0.47	0.85
Mean				3.88	2.41	0.48	0.49	0.90

*A* mean number of alleles per locus, *Ae* effective number of alleles, *He* expected heterozygosity, *Ho* observed heterozygosity, *I* Shannon–Weaver information index.

We also used Monmonier’s maximum difference algorithm to perform a genetic barrier prediction analysis for 14 populations from Guangxi, revealing five predicted barriers to gene flow (Supplementary Fig. S1). The You River-Yu River-Xun River formed a primary barrier that isolated the populations into two parts. Five populations (R_GX1, R_GX2, R_GX3, R_GX4, and R_GX5) were from the Nanliu River (Fig. 1b) but were a long distance apart. They were isolated from the other populations by the Darong and Liuwan Mountains. R_GX6, located in Fangchenggang, was isolated by the Gutong Mountain. R_GX9 belonged to Baise City and was distributed in small areas along the You River. R_GX13 was located in the northernmost area of rice distribution and was isolated by Shanzhao Ling and the Liu River.

Dongxiang County is recognized as the northernmost habitat of *O. rufipogon*, and Anjiashan and Shuitaoshu are the only two sites in Dongxiang where *O. rufipogon* is found. Four populations were surveyed to gain a more complete understanding of the genetic structure of *O. rufipogon* populations in Dongxiang. At the Anjiashan site, the primary in situ conservation site for *O. rufipogon* in China, a relatively large population is divided by a concrete wall that was constructed in the 1980s. DXP1, DXP2 and DXP3 are separate populations that are located close together and have been isolated by the concrete wall from the outside. DXP2 is located in the southeast, DXP3 in the northwest, and DXP1 in the middle. DXP4 is from Shuitaoshu and is further isolated by a hill (Fig. 1c).

We first investigated the genetic structures of the three natural populations from Anjiashan (DXP1, DXP2, and DXP3). The mean *H*_E_ estimates for DXP1, DXP2, and DXP3 were 0.47, 0.39 and 0.41, respectively, indicating that DXP1 had the highest genetic variation. However, the values of *H*_T_, *D*_ST_, and *G*_ST_ were 0.47, 0.050, and 0.098, suggesting that there was little differentiation among the three populations after more than 20 years of isolation by the concrete wall.

Next, DXP4 was included in the genetic structure analysis, together with the other three populations. The mean *D*_ST_ was 0.06, indicating that there was little genetic variation among the populations. The genetic differentiation over loci assessed by *G*_ST_ (0.12) was slightly higher than *D*_ST_, but it nonetheless indicated that there was minimal differentiation among the Dongxiang populations. The genetic similarity of all individuals from the four populations was 0.74, highlighting their close genetic relationship. Based on these analyses, the four Dongxiang populations can be considered a single population when collecting samples for ex situ conservation and the establishment of in situ conservation sites.

## Discussion

We examined *O. rufipogon* population differentiation at the national, provincial, and local levels using SSR markers. *F*_ST_ calculations showed that almost half of the total variation occurred among populations. Previously, Zhou^12^ used SSR markers to investigate twelve Chinese wild rice populations from four provinces and found high genetic differentiation among them (*R*_ST_ = 0.52). Zheng^16^ analyzed the sequences of seven chloroplastic and nuclear loci and found that pairwise *F*_ST_ values between *O. rufipogon* populations at the nuclear loci ranged from 0.3175 to 0.5748. The AMOVA and pairwise *F*_ST_ results in the present study provide further evidence for relatively high genetic differentiation and corroborate previous results. Zhou^12^ concluded that population isolation caused by habitat fragmentation increased genetic differentiation by increasing the frequency of inbreeding and clonal growth. Previous studies have reported *indica*-like and *japonica*-like differentiation in the *O. rufipogon* population^12,16,17^. Wang^18^ also suggested that spatial or physical isolation and local adaptation may contribute to population differentiation within this species. The *F*_IS_ of *O. granulata* was 0.402, suggesting that most populations deviated from Hardy–Weinberg expectation within populations and were deficient in heterozygotes. The *F*_ST_ of *O. granulata* was 0.859, indicating that 85.9% of the total genetic variation existed among populations^19^. For *O. officin*alis, *F*_IS_ was 0.899 and *F*_ST_ was 0.882^20^. Compared with *O. officinalis* (selfing) and *O. granulata* (selfing)*, O. rufipogon* has higher heterozygosity and greater genetic distances between populations. The value of *F*_IS_ in our result was varied from -0.49 to 0.44, indicating that the diversity of mating system among *O. rufipogon* populations was very high. The mean value of *F*_ST_ was 0.44, and it varied from 0.25 to 0.59, indicating that there was substantial genetic variation among populations. The diversity patterns of species in the genera *Leavenworthia*^21^, *Lycopersicon*^22^ and *Miscanthus*^23^ with different mating systems are similar. The accumulation of mutations from generation to generation as a result of asexual reproduction may also contribute to high heterozygosity in *O. rufipogon*. In outbreeding species, a decrease in recombination rates is observed in certain regions of the genome, especially around centromeres^24^. On the contrary, in species with a high level of inbreeding, the rarity of double heterozygotes results in lowered effective recombination rates in the whole genome. Therefore, it is expected that both hitch-hiking and background selection will strongly affect genetic variability in inbreeding species^24^.

Here, we identified a triangular area, including northern Hainan, southern Guangdong, and southwestern Guangxi, as the genetic diversity center of *O. rufipogon* in China. Previously, a genetic diversity center for *O. rufipogon* in south China, including Guangdong and Guangxi provinces, was proposed based on random amplified polymorphic DNA analysis^13^, allozyme analysis^9^, and SSR data^12^. However, our samples were collected according to a more systematic sampling strategy^25^ in which sampled individuals were at least 12 m apart, and approximately 30 individuals were sampled from each population to encompass at least 95% of its genetic diversity. Our genetic diversity center included one population from Hainan that was not included in the previous diversity center. Gao^11^ found that, like Guangdong and Guangxi, Hainan also maintained higher levels of microsatellite diversity. Wang^18^ also found that Hainan ranked first in China with respect to its gene diversity index and gene richness. It is reasonable that the genetic diversity center includes Hainan because it has appropriate annual temperatures (16–23 °C) and precipitation (approximately 1,400 mm), as well as higher levels of outbreeding and diversity of ecological habitats^26^. In addition, populations in our proposed diversity center had more private alleles than did populations from other areas. Although the diversity of phenotypes in populations within and outside the proposed diversity center should be documented^27,28^ and compared with molecular data, our SSR data strongly suggest that the proposed triangular area is the diversity center of *O. rufipogon* in China. Southeastern China has generally enjoyed relative tectonic stability since the late Tertiary^29^, with perhaps the single exception of its two large islands. The high percentage of endemics in this region^30^ suggests that central and south China has played a significant role both as a center of survival but also as a center of plant differentiation and evolution during the Quaternary^31^. This may explain why the center of *O. rufipogon* genetic diversity is located in Southeastern China.

*O. rufipogon* is the most important genetic resource for rice breeding and the most endangered wild rice species in China; its collection and conservation are therefore increasingly important. At the national level, a region in southern China whose populations have higher genetic variation and more private alleles is likely to be the genetic diversity center of *O. rufipogon*. More valuable genes may exist in populations from this area, and its gene pool may be more useful for future variety improvement and biotechnology applications. Therefore, attention should be focused on *O. rufipogon* from this area for both the construction of in situ conservation sites and the collection of ex situ samples. However, populations outside the genetic center are also important for conservation: almost half of the genetic diversity and 32 out of 76 private alleles existed in these populations. Indeed, populations with relatively low genetic diversity may contain unique alleles that are absent from the diversity center (Table 5). More than 40% of the variation between populations also supported the conclusion that populations from outside the diversity center should receive attention. Based on our analysis of populations across the country, populations in the genetic diversity center should be given first priority when developing national strategies for *O. rufipogon* conservation. Nonetheless, populations in regions with special ecological conditions, such as unique soils, climates, or valley locations, should also be considered.

**Table 5:** Summary of the private SSR alleles detected inside and outside the genetic diversity center.

Group	No. of genotypes	Private alleles	
		No	Richness
Diversity center	196	44	0.22
Non-diversity center	184	32	0.17
Total	228	76	0.33
Chi-square test		20.2***	

***P < 0.001.

Based on the genetic structure analysis of wild rice in Guangxi and Jiangxi, local environmental conditions appear to have influenced gene flow to a greater extent than geographic distance during population genetic evolution. The geography of R_GX9 belonged region is unique, consisting of valleys surrounded by mountains, and the spread of wild rice has therefore been curtailed. The annual minimum temperature of the northernmost area of rice distribution where R_GX13 was located is often below 0 ℃ in this region. The unique local climate and geography have shaped the distinct characteristics of wild rice from the northern mountains. Moreover, The finding that populations from the lower and middle regions of a river contained more genetic variation than those from the upstream regions suggests that conservation efforts should be focused on the downstream populations. Similar genetic diversity results have been reported for plants growing in several important watersheds in China, including *Myricaria laxiflora* from the Changjiang River in the Three Gorges Region^32^ and *Sophora moorcroftiana* along the Yarlung Zhangbo River^33^.

Smaller population sizes that result from habitat fragmentation may lead to a loss of genetic variation through genetic drift, thereby increasing population differentiation^34^. However, we found that population divergence was more significantly correlated with environmental conditions than with geographic location and isolation by distance. Populations from similar ecosystems showed less genetic differentiation, and local environmental conditions rather than geographic distance appeared to have influenced gene flow during population genetic evolution. These results are consistent with the recently developed maximum genetic diversity (MGD) theory of molecular evolution^35,36^, which predicts that similar environments will select for similar genetic variants, regardless of geographic distance^37^. Environmental factors such as historical habitat fragmentation and local adaptation can cause divergence^38^, and adaptation to local conditions rather than simple geographic isolation appears to have driven *O. rufipogon* population differentiation. Our results suggest that ex situ sampling of multiple populations from similar ecosystems should not be a priority because such populations tend to be genetically similar even when they are separated by large distances. We should therefore reduce the number of samples for ex situ conservation collection to avoid duplication, no matter how far apart they are.

## Methods

### Population sampling

To analyze population genetic structure at the national level, 628 *O. rufipogon* accessions from 20 populations were collected at locations from 18° N to 28° N latitude. The locations of all 20 populations are shown in Fig. 1a. The sampling region covered all *O. rufipogon*’s natural distribution areas^7^ and spread across six provinces in southern China, including Hainan, Guangdong, Guangxi, Fujian, Hunan, and Jiangxi (Table 1). At each latitude, one to four representative populations were selected for analysis. Samples from the N_JX1 population in Jiangxi were collected from both Anjiashan and Shuitaoshu, as these are the only two locations in Jiangxi where *O. rufipogon* is found. Likewise, the N_FJ1 population is the only existing *O. rufipogon* population in Fujian. The N_JX1 and N_HuN1 populations are located at the northern boundary of *O. rufipogon*’s range in China. We expanded our sampling in Guangxi because this province contains the largest population of wild rice, and 380 accessions from 14 populations were collected from this province (Table 4). These samples included individuals from the most northern (R_GX14), southern (R_GX3, R_GX4, R_GX5), and western populations (R_GX7) in the province (Fig. 1b). Within each population, individuals were randomly collected at a distance of at least 5 m from one another to avoid collecting samples from a single genet.

### DNA isolation and polymerase chain reaction

Genomic DNA was extracted using the CTAB method according to the protocol of Edwards^39^. The quality and quantity of DNA were assessed on 0.8% agarose gels. DNA concentrations were determined using an ultraviolet spectrophotometer, and the solutions were then diluted to 20 ng/μL with a Tris–EDTA buffer. PCR amplifications were performed with a 5700 thermocycler (PE Applied Biosystems, USA). The PCR reaction in a total volume of 20 μl consisted of 100 mmol/L Tris–HCl, 1 U Taq polymerase, 2.5 mmol/L MgCl_2_, 2.5 mmol/L dNTPs, 4 μmol/L forward and reverse SSR primers, and 100 ng DNA. The PCR program was 5 min at 94 °C, followed by 35 cycles of 1 min at 94 °C, 50 s at 56–61 °C, and 1 min at 72 °C, with 10 min at 72 °C for the final extension. PCR products were mixed with 6 μl of loading buffer, denatured at 95 °C for 5 min, and separated in 6% polyacrylamide denaturing gels (38 × 30 × 1 cm^3^). The banding patterns were visualized according to the manufacturer’s instructions (Promega, USA).

### Statistical analysis

The original SSR data were preprocessed using DataFormater 2.7^40^, which transformed SSR data to readable input files for STRUCTURE, PowerMarker, Tassel, GENEPOP and POPGENE. Micro-Checker 2.2.3 was used to check for scoring errors and null alleles^41^. Based on solid foundation on study of *O. rufipogon* diversity, we selected the markers without null alleles for analysis^12,14,42–45^.We used POPGENE 1.31^46^ and GENEPOP 3.4^47^ to calculate the genetic diversity parameters: the mean number of alleles per locus (*A*), the effective number of alleles (*A*_E_), the Shannon–Weaver information index (*I*), the observed heterozygosity (*H*_O_), and the expected heterozygosity (*H*_E_)^48,49^. Deviation from Hardy–Weinberg equilibrium and population differentiation were assessed at each locus across all populations using *F* statistics, including the fixation index within populations (*F*_IS_), the fixation index across all populations (*F*_IT_) and the gene differentiation index (*F*_ST_)^50^. STRUCTURE was used to infer genetic clusters (*K*) with the model-based clustering method^51^. We assessed *K* values from 2 to 9 by performing ten independent runs for each *K* value, and the model was run with a 10,000 burn-in period and 100,000 Monte Carlo Markov chain repetitions. CLUMPP version 1.1^52^ was used to obtain the optimal clusters for each *K*. The relationships between populations were assessed by Nei’s^53^ standard genetic distance using the Unweighted Pair Group Method with Arithmetic Mean (UPGMA) in PowerMarker^54^. Principal component analysis (PCA) was performed using Tassel 3.0 (https://www.maizegenetics.net/tassel) to summarize the major patterns of variation. Analysis of molecular variance (AMOVA) was performed using Arlequin 3.11^55^, and Mantel 2.0^56^ was used to assess whether the data fit the hypothesis of isolation by distance, which predicts a significant relationship between geographic distance and genetic distance. A genetic barrier analysis was performed to suggest historical barriers to gene flow among or between collection sites using BARRIER^57^ (version 2.2, Syracuse University, USA) with Monmonier’s maximum difference algorithm, which takes the geographic coordinates and genetic distance (GD) of each population as inputs.

The presence of private alleles in each population and in groups within and outside the genetic diversity center were assessed, and the richness of private SSR alleles was defined as the average number of private alleles per genotype for each population.

## Supplementary information

Supplementary Information.
