# Supplementary material for: Conservation recommendations for Oryza rufipogon Griff. in China based on genetic diversity analysis
Source: Sci Rep. 2020 Sep 1;10:14375. doi: 10.1038/s41598-020-70989-w (PMC7462988; doi:10.1038/s41598-020-70989-w)
Supplement: Supplementary file 1 — Supplementary Information. [file 41598_2020_70989_MOESM1_ESM.pdf]

Supplementary Information

Conservation Recommendations for *Oryza rufipogon* Griff. in China Based on Genetic Diversity Analysis

Junrui Wang[1, †], Jinxia Shi[2, †], Sha Liu[1], Xiping Sun[3], Juan Huang[1, 4], Weihua Qiao[1,5], Yunlian Cheng[1], Lifang Zhang[1], Xiaoming Zheng[1,5]*, Qingwen Yang[1.5]*

[1] Institute of Crop Sciences, Chinese Academy of Agricultural Sciences, China; wangjunrui0202@foxmail.com (J.W.); 906036056@qq.com (S.L.); qiaoweihua@caas.cn (W.Q.); chengyunlian@caas.cn (Y.C.); zhanglifang@caas.cn (L.Z.)
[2] Shanghai Normal University, China; jinxiashi@shnu.edu.cn (J.S.)
[3] Shanxi Agricultural University, China; sxpljj@126.com (X.S.)
[4] Institute of Rice Research, Guangxi Academy of Agricultural Sciences, China; 554338363@qq.com (J.H.)
[5] Agricultural Science and Technology Innovation Program/Crop Germplasm Resources Preservation and Sharing Innovation Team, China
[†] These authors contributed equally to this work.
* Correspondence: yangqingwen@caas.cn (Q.Y.); zhengxiaoming@caas.cn (X.Z.)

Figure

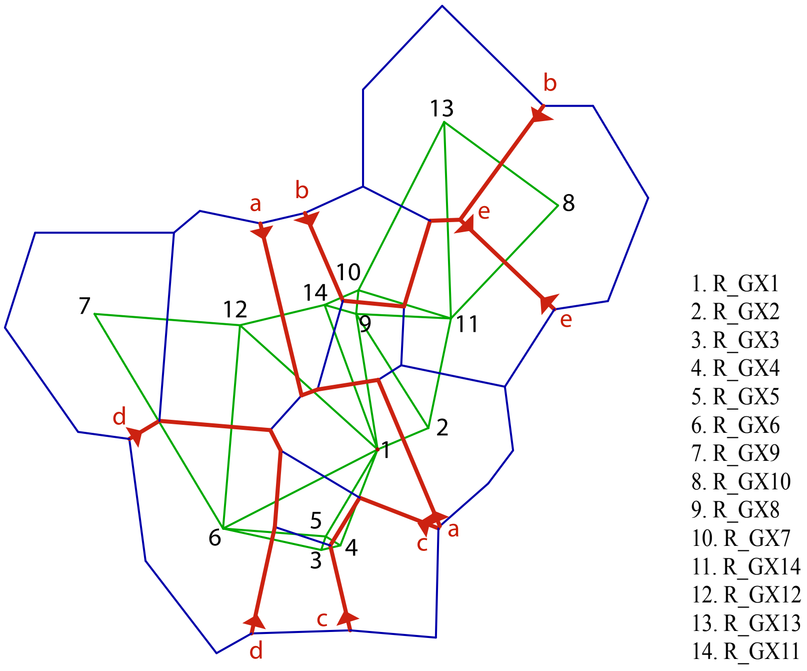

Figure S1: Genetic barriers predicted by BARRIER of populations from Guangxi province. The genetic barriers are shown in bold lines with arrows. Lines a, b, c, d, and e indicated genetic barriers.

Tables

Table S1 Information of SSR primer used in this study.

Locus	Chromosome	Forward primer	Reverse primer
RM129	1	TCTCTCCGGAGCCAAGGCGAGG	CGAGCCACGACGCGATGTACCC
RM212	1	CCACTTTCAGCTACTACCAG	CACCCATTTGTCTCTCATTATG
RM240	2	CCTTAATGGGTAGTGTGCAC	TGTAACCATTCCTTCCATCC
RM250	2	GGTTCAAACCAAGCTGATCA	GATGAAGGCCTTCCACGCAG
RM7	3	TTCGCCATGAAGTCTCTCG	CCTCCCATCATTTCGTTGTT
RM282	3	CTGTGTCGAAAGGCTGCAC	CAGTCCTGTGTTGCAGCAAG
RM131	4	TCCTCCCTCCCTTCGCCCACTG	CGATGTTCGCCATGGCTGCTCC
RM252	4	TTCGCTGACGTGATAGGTTG	ATGACTTGATCCCGAGAACG
RM161	5	TGCAGATGAGAAGCGGCGCCTC	TGTGTCATCAGACGGCGCTCCG
RM173	5	CCTACCTCGCGATCCCCCCCTC	CCATGAGGAGGAGGCGGCGATC
RM115	6	TTGCCGCAGTGGCCGTTACCAC	AGGAGGCGGCGGAAATGGAAGG
RM345	6	ATTGGTAGCTCAATGCAAGC	GTGCAACAACCCCACATG
RM118	7	CCAATCGGAGCCACCGGAGAGC	CACATCCTCCAGCGACGCCGAG
RM320	7	CAACGTGATCGAGGATAGATC	GGATTTGCTTACCACAGCTC
RM152	8	GAAACCACCACACCTCACCG	CCGTAGACCTTCTTGAAGTAG
RM264	8	GTTGCGTCCTACTGCTACTTC	GATCCGTGTCGATGATTAGC
RM257	9	CAGTTCCGAGCAAGAGTACTC	GGATCGGACGTGGCATATG
RM242	9	GGCCAACGTGTGTATGTCTC	TATATGCCAAGACGGATGGG
RM216	10	GCATGGCCGATGGTAAAG	TGTATAAAACCACACGGCCA
RM271	10	TCAGATCTACAATTCCATCC	TCGGTGAGACCTAGAGAGCC
RM120	11	CACACAAGCCCTGTCTCACGACC	CGCTGCGTCATGAGTATGTA
RM287	11	TTCCCTGTTAAGAGAGAAATC	GTGTATTTGGTGAAAGCAAC
RM247	12	TAGTGCCGATCGATGTAACG	CATATGGTTTTGACAAAGCG
RM277	12	CGGTCAAATCATCACCTGAC	CAAGGCTTGCAAGGGAAG

Table S2 Genetic diversity parameters of twenty populations in China based on SSR.

						F-Statistic			F	*t*
Name	*A*	*Ae*	*I*	*He*	*Ho*	*Fis*	*Fit*	*Fst*		
RM23	10.00	6.92	2.05	0.86	0.54	-0.20	0.40	0.50	0.37	0.46
RM212	13.00	4.84	1.77	0.79	0.59	-0.39	0.22	0.44	0.26	0.59
RM250	16.00	3.59	1.78	0.72	0.78	-0.45	0.03	0.33	-0.08	1.17
RM154	15.00	10.19	2.48	0.90	0.45	0.08	0.51	0.47	0.50	0.33
RM16	13.00	4.69	1.89	0.79	0.79	-0.49	0.04	0.36	-0.01	1.02
RM282	10.00	2.86	1.49	0.65	0.69	-0.41	-0.06	0.25	-0.06	1.13
RM280	16.00	4.65	2.01	0.78	0.58	-0.23	0.27	0.41	0.26	0.58
RM349	10.00	7.26	2.07	0.86	0.66	-0.23	0.36	0.48	0.23	0.62
RM267	14.00	6.98	2.10	0.86	0.48	-0.16	0.40	0.48	0.44	0.39
RM334	16.00	6.82	2.12	0.85	0.76	-0.35	0.18	0.39	0.10	0.81
RM253	23.00	13.59	2.78	0.93	0.69	-0.32	0.31	0.48	0.26	0.59
RM345	7.00	4.68	1.72	0.79	0.54	-0.28	0.28	0.44	0.46	0.37
RM336	22.00	15.41	2.85	0.94	0.88	-0.31	0.08	0.29	0.06	0.89
RM125	11.00	4.97	1.79	0.80	0.58	-0.28	0.37	0.51	0.27	0.57
RM72	19.00	11.76	2.62	0.92	0.59	-0.04	0.42	0.45	0.35	0.48
RM331	12.00	4.16	1.66	0.76	0.34	0.05	0.51	0.49	0.55	0.29
RM201	11.00	5.95	1.94	0.83	0.48	-0.11	0.49	0.54	0.43	0.40
RM278	15.00	5.87	2.11	0.83	0.46	-0.09	0.49	0.53	0.45	0.38
RM244	7.00	2.80	1.13	0.64	0.15	0.44	0.77	0.59	0.76	0.13
RM216	14.00	5.82	2.04	0.83	0.55	-0.17	0.31	0.41	0.33	0.50
RM229	18.00	11.36	2.61	0.91	0.68	-0.21	0.26	0.39	0.25	0.60
RM287	16.00	6.16	2.18	0.84	0.66	-0.32	0.22	0.41	0.21	0.65
RM17	13.00	7.89	2.27	0.87	0.62	-0.20	0.36	0.47	0.29	0.55
RM247	19.00	8.17	2.45	0.88	0.47	-0.13	0.45	0.51	0.47	0.36
Mean	14.17	6.97	2.08	0.83	0.58	-0.20	0.32	0.44	0.30	0.58

A: mean number of alleles per locus; *Ae*: effective number of alleles;*I*: ShannoN_Weaver information index; *He*: expected heterozygosity; *Ho*: observed heterozygosity; *Fis*: fixation index within populations; *Fit*: fixation index across all populations; *Fst*: gene differentiation index and F: fixation index.

Table S3 Pairwise genetic distance of 20 populations in China based on SSR.

	N_HN1	N_HN2	N_HN3	N_HN4	N_HN5	N_GD1	N_GX2	N_GD2	N_GD5	N_GX1	N_GD3	N_GD4	N_GX3	N_GX4	N_GD6	N_GD7	N_GX5	N_FJ1	N_HuN1
N_HN2	0.55*																		
N_HN3	0.54*	0.57*																	
N_HN4	0.52*	0.54*	0.53*																
N_HN5	0.41*	0.42*	0.44*	0.41*															
N_GD1	0.58*	0.52*	0.58*	0.51*	0.37*														
N_GX2	0.38*	0.39*	0.43*	0.36*	0.29*	0.33*													
N_GD2	0.42*	0.45*	0.47*	0.38*	0.31*	0.37*	0.25*												
N_GD5	0.57*	0.51*	0.58*	0.54*	0.39*	0.53*	0.37*	0.41*											
N_GX1	0.47*	0.46*	0.48*	0.41*	0.34*	0.42*	0.31*	0.33*	0.41*										
N_GD3	0.45*	0.45*	0.49*	0.42*	0.27*	0.36*	0.25*	0.26*	0.39*	0.29*									
N_GD4	0.56*	0.54*	0.56*	0.51*	0.38*	0.52*	0.33*	0.37*	0.51*	0.40*	0.30*								
N_GX3	0.44*	0.45*	0.47*	0.42*	0.34*	0.43*	0.32*	0.32*	0.43*	0.34*	0.30*	0.41*							
N_GX4	0.50*	0.49*	0.53*	0.47*	0.33*	0.47*	0.32*	0.33*	0.44*	0.34*	0.27*	0.42*	0.33*						
N_GD6	0.65*	0.58*	0.63*	0.56*	0.46*	0.59*	0.40*	0.42*	0.56*	0.43*	0.38*	0.53*	0.43*	0.37*					
N_GD7	0.48*	0.49*	0.52*	0.47*	0.37*	0.45*	0.35*	0.34*	0.46*	0.39*	0.31*	0.46*	0.36*	0.35*	0.40*				
N_GX5	0.53*	0.52*	0.57*	0.52*	0.41*	0.52*	0.35*	0.37*	0.49*	0.42*	0.35*	0.49*	0.40*	0.39*	0.46*	0.36*			
N_FJ1	0.58*	0.56*	0.59*	0.50*	0.40*	0.53*	0.37*	0.35*	0.53*	0.42*	0.36*	0.51*	0.38*	0.39*	0.52*	0.42*	0.45*		
N_HuN1	0.49*	0.51*	0.51*	0.47*	0.36*	0.46*	0.33*	0.35*	0.46*	0.39*	0.30*	0.45*	0.37*	0.36*	0.45*	0.36*	0.38*	0.41*	
N_JX1	0.51*	0.49*	0.50*	0.47*	0.37*	0.48*	0.37*	0.37*	0.49*	0.43*	0.35*	0.48*	0.37*	0.38*	0.44*	0.35*	0.35*	0.45*	0.32*

*P<0.001

Table S4 Genetic distance of 14 populations in Guangxi based on SSR.

Region	R_GX1	R_GX2	R_GX3	R_GX4	R_GX5	R_GX6	R_GX9	R_GX10	R_GX8	R_GX7	R_GX14	R_GX12	R_GX13	R_GX11
R_GX1	1													
R_GX2	0.72	1												
R_GX3	0.65	0.63	1											
R_GX4	0.67	0.55	0.6	1										
R_GX5	0.56	0.51	0.46	0.62	1									
R_GX6	0.62	0.51	0.48	0.53	0.58	1								
R_GX9	0.61	0.53	0.43	0.52	0.47	0.63	1							
R_GX10	0.54	0.5	0.41	0.49	0.52	0.62	0.6	1						
R_GX8	0.46	0.33	0.32	0.37	0.38	0.39	0.39	0.4	1					
R_GX7	0.64	0.48	0.49	0.54	0.49	0.59	0.61	0.57	0.69	1				
R_GX14	0.57	0.46	0.45	0.54	0.44	0.46	0.58	0.58	0.49	0.63	1			
R_GX12	0.48	0.43	0.38	0.43	0.45	0.52	0.49	0.51	0.36	0.5	0.46	1		
R_GX13	0.56	0.45	0.48	0.52	0.49	0.58	0.57	0.63	0.38	0.54	0.57	0.58	1	
R_GX11	0.59	0.6	0.46	0.54	0.5	0.62	0.72	0.68	0.36	0.61	0.53	0.5	0.59	1
